# Phenotype and Tissue Expression as a Function of Genetic Risk in Polycystic Ovary Syndrome

**DOI:** 10.1371/journal.pone.0168870

**Published:** 2017-01-09

**Authors:** Cindy T. Pau, Tim Mosbruger, Richa Saxena, Corrine K. Welt

**Affiliations:** 1 Reproductive Endocrine Unit, Massachusetts General Hospital, Boston, Massachusetts, United States of America; 2 Huntsman Cancer Institute Bioinformatics, University of Utah, Salt Lake City, Utah, United States of America; 3 Department of Anaesthesiology and Center for Human Genetics, Massachusetts General Hospital, Boston, Massachusetts, United States of America; 4 Division of Endocrinology, Metabolism and Diabetes, University of Utah, Salt Lake City, Utah, United States of America; John Hopkins University School of Medicine, UNITED STATES

## Abstract

Genome-wide association studies and replication analyses have identified (n = 5) or replicated (n = 10) DNA variants associated with risk for polycystic ovary syndrome (PCOS) in European women. However, the causal gene and underlying mechanism for PCOS risk at these loci have not been determined. We hypothesized that analysis of phenotype, gene expression and metformin response as a function of genotype would identify candidate genes and pathways that could provide insight into the underlying mechanism for risk at these loci. To test the hypothesis, subjects with PCOS (n = 427) diagnosed according to the NIH criteria (< 9 menses per year and clinical or biochemical hyperandrogenism) and controls (n = 407) with extensive phenotyping were studied. A subset of subjects (n = 38) underwent a subcutaneous adipose tissue biopsy for RNA sequencing and were subsequently treated with metformin for 12 weeks with standardized outcomes measured. Data were analyzed according to genotype at PCOS risk loci and adjusted for the false discovery rate. A gene variant in the *THADA* locus was associated with response to metformin and metformin was a predicted upstream regulator at the same locus. Genotype at the *FSHB* locus was associated with LH levels. Genes near the PCOS risk loci demonstrated differences in expression as a function of genotype in adipose including *BLK* and *NEIL2* (*GATA4* locus), *GLIPR1* and *PHLDA1 (KRR1* locus). Based on the phenotypes, expression quantitative trait loci (eQTL), and upstream regulatory and pathway analyses we hypothesize that there are PCOS subtypes. *FSHB*, *FHSR* and *LHR* loci may influence PCOS risk based on their relationship to gonadotropin levels. The *THADA*, *GATA4*, *ERBB4*, *SUMO1P1*, *KRR1* and *RAB5B* loci appear to confer risk through metabolic mechanisms. The *IRF1*, *SUMO1P1* and *KRR1* loci may confer PCOS risk in development. The *TOX3* and *GATA4* loci appear to be involved in inflammation and its consequences. The data suggest potential PCOS subtypes and point to the need for additional studies to replicate these findings and identify personalized diagnosis and treatment options for PCOS.

## Introduction

Polycystic ovary syndrome (PCOS) is the most common endocrinopathy in reproductive age women, affecting 7–10% of this group. Cardinal features include irregular menstrual cycles, hyperandrogenism and polycystic ovarian morphology [1]. Obesity and insulin resistance are also common, along with increased risk of diabetes, metabolic syndrome and other cardiovascular diseases [2, 3]. Despite the detrimental impact of the disorder on women’s health, the etiology remains poorly understood.

Twin studies suggest that genetic influences explain over 70% of PCOS pathogenesis [4]. Therefore, genome-wide association studies have been performed to uncover DNA variants associated with PCOS [5–9]. There are now 16 published variants that mark loci associated with PCOS in women of Han Chinese and European ethnicity. It is not clear which variants in the loci are causal for increased risk of PCOS nor which genes in the loci are marked by the genotyped variants. Insights have been gained previously through association with quantitative phenotype traits and PCOS subphenotypes [7, 9, 10]. Insight has also been gained through examination of gene expression in tissues of interest in other diseases, including type 2 diabetes [11–14].

We therefore hypothesized that the relationship between PCOS risk loci and quantitative phenotypic traits would illuminate the underlying PCOS features affected at each locus. We also hypothesized that gene expression and associated patterns would identify candidate genes and pathways that could provide insight into the risk mechanism at the loci. The data suggest relationships between PCOS risk variants and gonadotropins, reproductive, metabolic and developmental pathways.

## Results

### Phenotype/Genotype Analysis

**Fig 1:**
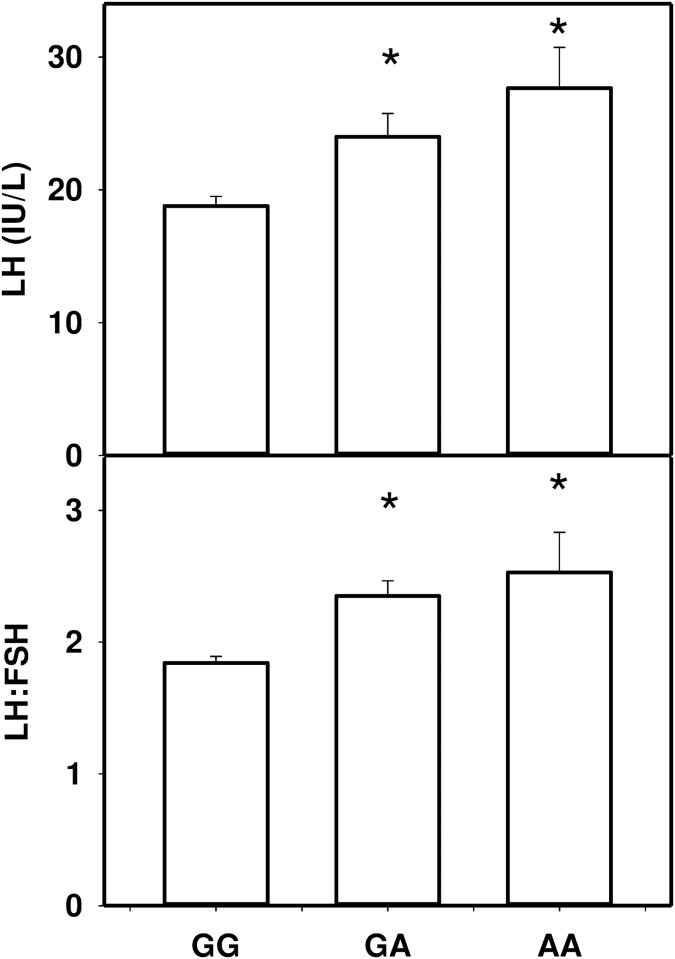
LH and the LH:FSH ratio as a function of genotype at rs11031006 in PCOS and control subjects. *p<0.05.

LH levels (genotype GG 18.8±0.7, GA 24.0±1.8, AA 27.7±3.1 IU/L; p = 1.13x10^-5^) and the LH:FSH ratio (GG 1.8±0.05, GA 2.4±0.1, AA 2.5±0.3; p = 1.1X10^-9^) were associated with genotype at rs11031006, near the *FSHβ* gene (Fig 1). Other phenotypic traits were not associated with genotype except as previously reported [15].

### Gene Expression

The number of reads was 30.3X10^6^±4.9 X10^6^, uniquely aligned reads was 23.5X10^6^±4.5 X10^6^ (77±8%), and the number of exonic reads was 9.6X10^6^±3.5 X10^6^ (40±11% of the unique reads). Skin and fat RNA expression clusters did not overlap and were therefore analyzed separately. Four samples did not cluster with their respective tissue types and were removed from the analysis (3 fat and 1 skin).

In adipose, *BLK* demonstrated increased expression (3.8±1.2-fold, p_adj_ = 0.0005) and *NEIL2* nominally decreased expression (-0.8±0.3-fold, p_adj_ = 0.057) at the rs804279 (*GATA4/NEIL2*) locus. *GLIPR1* (2.7±0.7-fold, p_adj_ = 1.2X10^-5^) and *PHLDA1* (3.1±0.5-fold, p_adj_ = 4.2X10^-12^) demonstrated increased expression at the rs1795379 (*KRR1*) locus. There was nominally decreased expression of *DENND1A* (-0.6±0.3-fold, p_nom_ = 0.02) at the rs10986105 (*DENND1A*) locus. There were no significant relationships when expression of genes within the same loci were examined in skin.

### Metformin Response

Subjects who carried the rs12478601-C PCOS risk variant were more likely to respond to metformin with lower basal glucose (χ^2^ = 9.1, p = 0.002; CC -4.15±2.22, CT -3.33±5.8, TT 2.03±7.24 mg/dL; p<0.003). There were no additional significant relationships between genotype and metformin response parameters. Metformin was predicted as an upstream regulator for *THADA* rs12478601 variant carriers (p = 3X10^-6^; S1 Table).

### Upstream Regulators

The top 5 predicted upstream regulators for each variant are shown in Table 1. The entire list of upstream regulators is shown in S1 Table.

**Table 1:** Top five upstream regulators predicted by pattern of gene expression as a function of genotype at polycystic ovary syndrome risk loci. Three variants, rs4385527 (*C9orf3*), rs2272046 (*HMG2A*) and rs2059807 (*INSR*), did not have data available.

				Growth Factors							Chemical or Endogenous Stimulator								Insulin Signaling				Chol Signaling		Immune and Inflammatory Regulators						Other
Variant	Chr	Nearest Gene	Tissue	*TGFB1*	*ERBB/ERBB2*	*PDGFBB*	*VEGF*	*IGF1*	*EGF*	*EREG*	FSH	Estradiol	Mifepristone	Dexamethasone	Drosperinone	Genestein	Tretinoin	Phorbol Esters	*INSR*	*GCG*	*PPARA*	*PPARG*	*SREBF1&2*	*SCAP*	*LPS*	*TNF*	*IL2*, *IL4*, *IL13*	*IFNG*	*Immunoglobulin*	*TCR*	
rs12478601	2	*THADA*	Fat	X	X																	X									
rs12478601	2	*THADA*	Skin									X		X							X		X								
rs2178575	2	*ErbB4*	Fat												X																X
rs2178575	2	*ErbB4*	Skin																												X
rs2268361	2	*FSHR*	Fat			X			X																	X					
rs2268361	2	*FSHR*	Skin	X			X																		X	X		X			
rs13405728	2	*LHCGR*	Skin	X			X										X									X		X			
rs13164856	5	*RAD50*	Skin							X																					X
rs804279	8	*GATA4/NEIL2*	Fat																								X		X	X	
rs10986105	9	*DENND1A*	Fat	X				X				X															X				X
rs2479106	9	*DENND1A*	Skin																												
rs11031006	11	*FSHB*	Skin										X																		X
rs11031005	11	*FSHB*	Fat														X								X	X	X				
rs705702	12	*RAB5B/SUOX*	Fat																						X		X		X	X	
rs705702	12	*RAB5B/SUOX*	Skin																		X	X	X	X							
rs2271194	12	*ErbB3*	Fat																						X		X				
rs1795379	12	*KRR1*	Fat	X	X																					X		X			X
rs4784165	16	*TOX3*	Fat	X																							X		X	X	
rs6022786	20	*SUMO1P1*	Fat								X								X												X
rs6022786	20	*SUMO1P1*	Skin											X						X			X								X

The most common upstream regulators included *TGFB1* (*THADA*, *FSHR*, *LHCGR*, *DENND1A*, *KRR1*, *TOX3*) and the immune and inflammatory regulators tumor necrosis factor, interleukins and interferon gamma (*THADA*, *FSHR*, *LHCGR*, *GATA4/NEIL2*, *DENND1A*, *ERBB3*, *KRR1*, *TOX3*). Other upstream regulators included mitochondrial genes and *AMPK* upstream at the *ERBB4* locus, important for energy production. Transcription factors *HOXA9*, *TP53* and *GATA2* were upstream regulators of *SUMO1P1* and *KRR1*, respectively. *ERBB2* was an upstream regulator of *THADA* and *KRR1*.

### Canonical Pathways

The expression patterns at the majority of the loci overlapped with several canonical pathways involved in the immune and inflammatory response (Table 2 and S2 Table). In addition, the expression pattern at rs12478601 in fat (*THADA* locus) overlapped with a canonical pathway involved in adipogenesis. In skin, canonical pathways included cholesterol biosynthesis pathways.

**Table 2:** Top five canonical pathways identified by gene expression patterns at each polycystic ovary syndrome genetic risk locus.

Variant	Candidate Gene(s)	Tissue	Ingenuity Canonical Pathways
rs1795379	KRR1	Fat	Actin Cytoskeleton Signaling
rs2479106	DENND1A	Skin	Acyl-CoA Hydrolysis
rs12478601	THADA	Fat	Adipogenesis pathway
rs4784165	TOX3	Fat	Agranulocyte Adhesion and Diapedesis
rs2178575	ERBB4	Fat	Allograft Rejection Signaling
rs2479106	DENND1A	Skin	Antigen Presentation Pathway
rs2268361	FSHR	Fat	Aryl Hydrocarbon Receptor Signaling
rs10986105	DENND1A	Fat	
rs11031006	FSHB	Skin	Atherosclerosis Signaling
rs2178575	ERBB4	Skin	
rs4784165	TOX3	Fat	
rs2178575	ERBB4	Fat	Autoimmune Thyroid Disease Signaling
rs2268361	FSHR	Skin	Axonal Guidance Signaling
rs13405728	LHCGR	Skin	
rs1795379	KRR1	Fat	
rs2479106	DENND1A	Skin	B Cell Development
rs705702	RAB5B/SUOX	Fat	B Cell Receptor Signaling
rs11031005	FSHB	Fat	
rs10986105	DENND1A	Fat	Basal Cell Carcinoma Signaling
rs705702	RAB5B/SUOX	Fat	CD28 Signaling in T Helper Cells
rs804279	GATA4/NEIL2	Fat	
rs13164856	RAD50	Skin	Ceramide Degradation
rs12478601	THADA	Skin	Cholesterol Biosynthesis I
rs12478601	THADA	Skin	Cholesterol Biosynthesis II (via 24,25-dihydrolanosterol)
rs12478601	THADA	Skin	Cholesterol Biosynthesis III (via Desmosterol)
rs11031005	FSHB	Fat	Complement System
rs2268361	FSHR	Skin	Ephrin Receptor Signaling
rs13405728	LHCGR	Skin	
rs1795379	KRR1	Fat	
rs13164856	RAD50	Skin	Epoxysqualene Biosynthesis
rs13164856	RAD50	Skin	ErbB Signaling
rs12478601	THADA	Fat	Ethanol Degradation II
rs2479106	DENND1A	Skin	Eumelanin Biosynthesis
rs705702	RAB5B/SUOX	Skin	Fatty Acid Activation
rs705702	RAB5B/SUOX	Skin	Fatty Acid β-oxidation I
rs6022786	SUMO1P1	Skin	FXR/RXR Activation
rs2271194	ERBB3	Fat	Glucocorticoid Receptor Signaling
rs13405728	LHCGR	Fat	
rs4784165	TOX3	Fat	Granulocyte Adhesion and Diapedesis
rs2178575	ERBB4	Fat	Hematopoiesis from Pluripotent Stem Cells
rs10986105	DENND1A	Fat	Hepatic Fibrosis / Hepatic Stellate Cell Activation
rs1795379	KRR1	Fat	
rs4784165	TOX3	Fat	
rs11031005	FSHB	Fat	HER-2 Signaling in Breast Cancer
rs2268361	FSHR	Fat	HGF Signaling
rs13405728	LHCGR	Fat	HMGB1 Signaling
rs4784165	TOX3	Fat	
rs705702	RAB5B/SUOX	Fat	iCOS-iCOSL Signaling in T Helper Cells
rs804279	GATA4/NEIL2	Fat	
rs13405728	LHCGR	Fat	IL-17A Signaling in Fibroblasts
rs11031005	FSHB	Fat	IL-3 Signaling
rs13405728	LHCGR	Fat	IL-6 Signaling
rs2268361	FSHR	Fat	JAK/Stat Signaling
rs6022786	SUMO1P1	Skin	Leucine Degradation I
rs804279	GATA4/NEIL2	Fat	Leukocyte Extravasation Signaling
rs2268361	FSHR	Skin	
rs10986105	DENND1A	Fat	LPS/IL-1 Mediated Inhibition of RXR Function
rs6022786	SUMO1P1	Skin	
rs6022786	SUMO1P1	Fat	LXR/RXR Activation
rs2178575	ERBB4	Skin	Mitochondrial Dysfunction
rs13405728	LHCGR	Skin	Molecular Mechanisms of Cancer
rs13164856	RAD50	Skin	NADH Repair
rs705702	RAB5B/SUOX	Fat	Natural Killer Cell Signaling
rs804279	GATA4/NEIL2	Fat	
rs12478601	THADA	Fat	Noradrenaline and Adrenaline Degradation
rs2178575	ERBB4	Skin	Oxidative Phosphorylation
rs2178575	ERBB4	Skin	p38 MAPK Signaling
rs2178575	ERBB4	Fat	Primary Immunodeficiency Signaling
rs2268361	FSHR	Fat	Proline Biosynthesis II (from Arginine)
rs2271194	ERBB3	Fat	
rs10986105	DENND1A	Fat	Prostanoid Biosynthesis
rs2268361	FSHR	Fat	Protein Citrullination
rs2271194	ERBB3	Fat	
rs2178575	ERBB4	Skin	
rs6022786	SUMO1P1	Fat	Pyridoxal 5'-phosphate Salvage Pathway
rs1795379	KRR1	Fat	Rac Signaling
rs11031006	FSHB	Skin	Retinoate Biosynthesis I
rs12478601	THADA	Fat	
rs2271194	ERBB3	Fat	Role of IL-17A in Psoriasis
rs2268361	FSHR	Skin	Role of Macrophages, Fibroblasts and Endothelial Cells in Rheumatoid Arthritis
rs13405728	LHCGR	Fat	
rs13405728	LHCGR	Skin	
rs2268361	FSHR	Skin	Role of Tissue Factor in Cancer
rs6022786	SUMO1P1	Fat	Salvage Pathways of Pyrimidine Ribonucleotides
rs2271194	ERBB3	Fat	Serine Biosynthesis
rs12478601	THADA	Fat	Sertoli Cell-Sertoli Cell Junction Signaling
rs6022786	SUMO1P1	Fat	Spermine and Spermidine Degradation I
rs13164856	RAD50	Skin	Sphingosine and Sphingosine-1-phosphate Metabolism
rs705702	RAB5B/SUOX	Skin	Stearate Biosynthesis I (Animals)
rs2479106	DENND1A	Skin	
rs6022786	SUMO1P1	Skin	
rs705702	RAB5B/SUOX	Skin	Superpathway of Cholesterol Biosynthesis
rs12478601	THADA	Skin	
rs12478601	THADA	Skin	Superpathway of Geranylgeranyldiphosphate Biosynthesis I (via Mevalonate)
rs705702	RAB5B/SUOX	Fat	T Cell Receptor Signaling
rs804279	GATA4/NEIL2	Fat	
rs13405728	LHCGR	Skin	Thrombin Signaling
rs11031006	FSHB	Skin	Thymine Degradation
rs11031006	FSHB	Skin	Trehalose Degradation II (Trehalase)
rs6022786	SUMO1P1	Skin	Triacylglycerol Biosynthesis
rs11031006	FSHB	Skin	Uracil Degradation II (Reductive)
rs11031005	FSHB	Fat	Virus Entry via Endocytic Pathways
rs6022786	SUMO1P1	Fat	Zymosterol Biosynthesis
rs705702	RAB5B/SUOX	Skin	γ-linolenate Biosynthesis II (Animals)

The expression pattern for rs2178575 (*ERBB4* locus) in skin overlapped with canonical pathways involved in mitochondrial dysfunction, oxidative phosphorylation and MAPK signaling. The expression pattern for r2268361 (*FSHR* locus), rs13405728 (LHCGR locus) and rs1795379 (*KRR1* locus) overlapped with a canonical pathway involved in axonal guidance. The expression pattern at two loci overlapped with canonical pathways involved in ErbB signaling (rs13164856; *IRF1* locus) and ErbB2 signaling (rs11031005; FSHB locus).

### Associated Networks

The expression pattern at rs12478601 (*THADA* locus) in skin and fat, rs804279 (*GATA4* locus) and rs1795379 (*KRR1* locus) in fat and rs2268361 (*FSHR* locus) in skin predicted involvement in reproductive diseases (Table 3). The expression pattern at rs11031005 (*FSHβ* locus) in fat predicted a relationship to endocrine function and the pattern for rs2178575 (*ERBB4* locus) in skin predicted a relationship to metabolic disease.

**Table 3:** Top five networks identified by gene expression patterns at each polycystic ovary syndrome genetic risk locus.

Variant	Chr	Nearest Gene	Tissue	Reproductive Disease	Endocrine Function	Dermatologic Disease	Lipid Biosynthesis or Metabolism	Embryonic Development	Cell Growth& Proliferation	Cell-Cell Signaling	Cell Function and Maintenance
rs12478601	2	*THADA*	Skin	X			X	X	X	X	
rs2178575	2	*ErbB4*	Fat	X	X			X	X		
rs2268361	2	*FSHR*	Skin	X			X	X	X	X	X
rs13405728	2	*LHCGR*	Skin	X			X		X	X	X
rs13164856	5	*RAD50*	Skin			X	X	X	X		
rs804279	8	*GATA4/NEIL2*	Fat	X		X			X	X	X
rs2479106	9	*DENND1A*	Skin	X				X			
rs11031005	11	*FSHB*	Fat		X		X	X	X	X	
rs705702	12	*RAB5B/SUOX*	Skin	X			X			X	X
rs1795379	12	*KRR1*	Fat			X		X	X		
rs6022786	20	*SUMO1P1*	Skin				X	X	X		X

All of the loci were associated with at least one network involved in cell processes, including cell-to-cell signaling, movement, cell function and maintenance, cell growth, proliferation and development and cell death and survival. In addition, 8 loci were associated with networks predicted to involve embryonic development (*THADA*, *ERBB4*, *FSHR*, *RAD50*, *DENND1A*, *FSHβ*, *KRR1*, *SUMO1P1*). Finally, 7 loci were associated with cholesterol or lipid biosynthesis (*THADA*, *FSHR*, *LHCGR*, *RAD50*, *FSHβ*, *RAB5B*, *SUMO1P1*).

## Discussion

The risk attributed to genetic loci associated with PCOS can be understood only through fine mapping to locate the causal risk allele and identification of the candidate genes affected. Phenotype, both baseline and interrogated, and expression data from adipose and skin of women with PCOS suggest candidate genes and pathways that predispose to PCOS at the genetic risk loci. Specifically, phenotype data from current and previous studies suggest risk in relation to gonadotropins and glucose metabolism. Interrogation with metformin points to a locus with type 2 diabetes risk. The expression data point to the importance of growth factors and inflammatory and immune pathways in upstream regulation and effector pathways including reproductive, endocrine, metabolism and inflammatory pathways. The differences in potential upstream regulators and networks affected in the case of each variant point to potentially differing etiologies for PCOS risk at the distinct risk loci.

The phenotype insights are most helpful to ascertain. The rs11031006 variant is associated with LH levels and the LH:FSH ratio, as we previously reported for the rs12294104, which is in linkage disequilibrium [7, 16]. The variant is located upstream of FSHβ and larger studies demonstrate that FSH levels are also suppressed in the presence of the variant [7, 17]. Therefore, the variant may act as an upstream suppression site lowering FSH levels, with the resulting free α subunit pairing with LHβ and increasing LH levels [18, 19]. FSHβ expression was not evaluable in adipose or skin and the effect of the variant could not be examined in these tissues. The expression pattern in relation to the variant did predict involvement in endocrine development and disease. Finally, the overlap between the expression pattern at the FSHβ locus and ERBB2 signaling is supported by the relationship between gonadotropins, estradiol and HER-2 upregulation in breast cancer patients [20].

The rs12478601-C variant, identified initially in women of Han Chinese ethnicity and replicated in European women, was found in the only PCOS risk locus that predicted any defined response to metformin [6, 21, 22]. An independent *THADA* locus has been extensively evaluated and is associated with a lower insulin sensitivity in one study and decreased beta cell responsiveness [23]. Further, the expression pattern overlaps with the canonical adipogenesis pathway. Taken together, the data point to a relationship between the *THADA* locus in PCOS and the independent causal association with insulin resistance and metabolism [7].

There were few variants associated with gene expression at the loci identified. In carriers of the rs804279-A risk allele (*GATA4* locus), B cell non-receptor Src tyrosine kinase expression (*BLK)* was increased, a gene responsible for B-cell receptor signaling and B-cell development. It is a susceptibility gene for multiple autoimmune phenotypes. Interestingly, *BLK* protein also stimulates insulin synthesis and secretion in response to glucose and enhances expression of β cell transcription factors [24]. One hypothesis would be that the increased expression is a response to insulin resistance in PCOS. *NEIL2* was nominally decreased at the same locus, a gene that plays a role in gene maintenance and protection from DNA mutagenesis and the inflammatory response [25]. Upstream regulators at the locus were also dominated by inflammatory and immune genes. Taken together, the increase in expression of an autoimmune risk gene and trend toward decreased expression of an inflammatory suppression gene may point to an inflammatory risk profile in PCOS.

At rs1795379 (*KRR1* locus), there was increased expression of *GLIPR1* and *PHLDA1*. *PHLDA1* is the pleckstrin homology like domain family A member 1, a proline-histidine rich nuclear protein that plays an important role in the anti-apoptotic effects of insulin-like growth factor-1 (IGF-1) [26]. Interestingly, *PHLDA1* is induced by IGF-1 and insulin upregulates IGF-1 receptors in conditions with insulin resistance, as in PCOS [27]. *GLIPR1*, or GLI pathogenesis related 1, has proapoptotic activity in prostate cancer cells, is expressed at high levels in the testes and may have a role in sperm-oocyte interactions [28]. The increased gene expression points to possible relationships to insulin resistance and fertility at the locus, as suggested by the overlap in expression as a function of the rs1795379 and pathways involved in reproduction.

A variant located in an intron of *DENND1A* demonstrated a nominal decrease in *DENND1A* expression although it was no longer significant after correction for multiple testing [10, 21]. Although rs2479106 was not initially replicated, it was the variant replicated in the recent meta-analysis GWAS of data from European women [8], but was not associated with expression and not in linkage disequilibrium with rs10986105. Upstream regulators of rs10986105 include several factors important in reproductive pathways. For example, JUN is induced by GnRH and TGFB1 is important for reproductive regulation and has been implicated in PCOS [29, 30].

Previous studies have demonstrated differential gene expression in adipose tissue at the *RAB5B* and *INSR* PCOS risk loci [31]. The gene expression patterns at these two loci did not replicate in the current study. The loci with significant expression data were found in new loci that were not assessed in the previous study [31].

The IPA upstream regulatory analytic identified upstream transcriptional regulators that can explain observed gene expression differences as a function of genotype at each locus. The IPA network analysis integrates upstream regulators with downstream effects that may explain what is happening upstream and how it influences a phenotype or functional outcome downstream. The most common upstream regulators belong to the immunomodulatory and inflammatory axis. Previous studies have examined the association between polycystic ovary syndrome risk and variants in 80 inflammatory genes and found no association [32]. However, the study did not examine pathways that may influence PCOS through PCOS risk loci identified by GWAS, as in the current study. The second most common upstream regulators included growth factors, notably *TGFB1*. *TGFB1* is important for reproductive regulation and has been implicated in PCOS [29, 30]. The networks affected are mainly related to cell growth, maintenance and embryonic development. These upstream regulators and networks may be specific to adipose and skin tissue sources. Adipose tissue and skin have previously been demonstrated to share the most *cis*-effects in the regulatory regions [12]. Study of additional tissues may provide differing expression patterns and further insight into the mechanism of risk at these PCOS loci.

The analysis pathways also suggest the hypothesis that these loci confer risk for PCOS through distinct mechanisms. The *FSHB*, *FSHR* and *LHCGR* loci influence PCOS risk based on their relationship to gonadotropin levels, as demonstrated in the current study and previous studies [7, 9, 15]. The *THADA*, *GATA4*, *ERBB4*, *SUMO1P1* and *KRR1* loci appear to confer risk through metabolic mechanisms. The *RAB5B* locus also appears to confer risk through metabolic mechanisms, particularly through glucose metabolism, as described previously [15]. *KRR1* and *DENND1A* expression patterns suggest reproductive function. The *IRF1*, *SUMO1P1* and *KRR1* loci may confer PCOS risk through a developmental mechanism. The *TOX3* and *GATA4* loci appear to be involved in inflammation and its consequences.

The main limitation is the small sample size for PCOS-specific adipose and skin tissue expression. These studies will need replication in additional, larger PCOS cohorts. Other limitations include the possibility that the tissues examined in the current study are not those that are critical for functional expression of all genes. We also used subcutaneous adipose, which may not reflect important expression differences in visceral adipose, although it has been demonstrated to be similar for a small number of relevant genes [33, 34] associated previously with metabolic risk in PCOS. The subcutaneous adipocytes are also larger in women with PCOS and had lower lipoprotein lipase activity, suggesting a qualitative abnormality [35]. Ideally, ovarian tissue and pituitary/hypothalamic tissue should be studied next. By examining only *cis*-eQTLs we are potentially missing *trans*-eQTL effects [12]. However, given the small sample size there is concern for overcalling relationships. Finally, we did not examine controls because the hypothesis addressed possible eQTL effects in women with PCOS.

Phenotype data demonstrate a strong relationship between the risk alleles and gonadotropin levels. The data also demonstrate candidate genes at two PCOS risk loci marked by *GATA4/NEIL2* and *KRR1*. Expression patterns as a function of genotype point to specific influences on PCOS risk at each locus spanning reproductive, metabolic and inflammatory pathways and response to metformin maps to a locus that has a metabolic profile. These data suggest the hypothesis that PCOS can be subdivided into differing risk patterns that may be able to pinpoint the underlying etiology as a function of genotype.

## Methods

### Study Subjects

Subjects were of European ethnicity, aged 18–45 years old and with PCOS defined by the NIH criteria, i.e. irregular menses (< 9 menstrual periods/yr) and clinical or biochemical hyperandrogenism (n = 427)[36]. Clinical hyperandrogenism was defined as a Ferriman Gallwey (FG) score greater than 9, the upper 95% confidence limit for the Boston-based control populations. Biochemical hyperandrogenism was defined as an androgen level greater than the 95 percent confidence limits in control subjects with regular, ovulatory menstrual cycles: testosterone >63 ng/mL (2.8 nmol/L), DHEAS >430 μg/dL (1.16 μmoL/L) or androstenedione levels >3.8 ng/mL (0.13 nmol/L). Subjects with non-classic congenital adrenal hyperplasia, hypothyroidism, elevated prolactin levels, diabetes, Cushing syndrome and primary ovarian insufficiency were excluded [37]. Control subjects (n = 407) consisted of women aged 18–45 years with regular menses, between 21 and 35 days, and no hyperandrogenism [37]. Subjects were on no hormonal medication for at least 3 months and no medications that influence insulin, inflammation, or lipid levels for at least one month. A subset of subjects, meeting all criteria for PCOS as described above, were also studied in a protocol in which metformin was administered (n = 38)[36].

### Protocol

All PCOS subjects were studied ≥10 days after their last menstrual period and after a 12 hour fast [37]. Subjects underwent a detailed history; physical exam including measurement of waist circumference at the umbilicus and hip circumference at the widest diameter; a pelvic ultrasound (Phillips, 5 MHz convex array transducer); and blood samples for DNA, lipids, glucose, insulin, gonadotropin and sex-steroid levels. LH and FSH levels were obtained at ten minute intervals to calculate an average gonadotropin concentration. Using data from blood samples collected every 10 min over 12 h in women with PCOS and ovulatory controls [38], we documented that the mean LH secretion from 12 h of frequent blood samples correlates well with the value obtained from the mean of three samples collected from 0800–0820 h (r = 0.92, p<0.01) [37].

The subset of 38 subjects with PCOS also underwent an intravenous glucose tolerance test (IVGTT), with glucose 0.3 g/kg administered at time 0 and regular human insulin 0.03 U/kg injected at 20 minutes [22]. They subsequently underwent a subcutaneous adipose biopsy under local anesthesia with 1% lidocaine. Adipose tissue and skin, which was saved for 16 subjects, were rinsed with sterile 0.9% NaCl solution, placed on ice and transported for immediate freezing in liquid nitrogen.

After the initial visit, subjects started treatment with metformin ER 500 mg per day, with the dose increasing by 500 mg every two weeks to a final dose of 1500 mg/day, which was administered for a total of 12 weeks. Subjects returned every two weeks for anthropomorphic measurements, estradiol and progesterone levels, and a pelvic ultrasound to monitor folliculogenesis. Subjects returned for additional visits if follicle size indicated impending ovulation. Compliance was determined by questioning at the biweekly visits. After 12 weeks of metformin ER 1500 mg/day, subjects were admitted to the Massachusetts General Hospital Clinical Research Center to repeat the study as outlined above. Plasma metformin levels were measured in the fasting state at the study conclusion before the final IV glucose tolerance test. Clinical parameters were previously reported [22].

The study was approved by the Partners Human Research Committee. All subjects provided written, informed consent.

### Genotyping, RNA Sequencing and Statistical Analysis

Patient DNA was isolated from whole blood according to manufacturer’s specifications (Qiagen, USA) and genotyped using the HumanOmniExpress BeadChip (Illumina, San Diego). Associations between five SNPs not previously examined in our data (Table 1 and S3 Table) and PCOS phenotypes were evaluated [7–9].

Linear regression using an additive genetic model was used to test for association of PCOS risk variants with 30 log-transformed quantitative traits in the combined sample of PCOS cases and controls in the discovery Boston cohort. A *p* value < 0.0003 was considered significant after Bonferroni correction for 12 evaluable gene variants (S3 Table) and 15 independent traits (FSH, LH, prolactin, 17-OH progesterone, testosterone, cholesterol, sex hormone binding globulin (SHBG), estradiol, blood pressure, pulse, thyroid stimulating hormone (TSH), body mass index (BMI), fasting glucose, fasting insulin and ovarian volume), with other variables highly correlated.

Associations between genetic variants and response to metformin were analyzed using Chi square [39] and one way ANOVA. To correct for multiple testing, a p value of 0.0021 was considered significant to correct for comparisons of 2 independent outcomes (change in baseline glucose and glucose mediated glucose disposal, and testosterone and ovulation) and 12 evaluable variants.

SNPs from all loci associated with PCOS in European and Chinese GWAS and available on the HumanOmniExpress BeadChip were examined in relation to adipose and skin expression [5–9].

Skin (n = 10) and adipose tissue (n = 33) were isolated, tissue homogenized and RNA extracted (RNeasy or RNeasy lipid kits, Qiagen, USA). RNA libraries were prepared using Illumina TruSeq strand RNA sample prep with RiboZero treatment and sequencing was performed using the Illumina HiSeq 50 cycle single-read sequencing.

Transcript annotations for hg19 (build 74) were downloaded from Ensembl. Splice junction sequences were generated using the USeq (v8.8.2) MakeTranscriptome application using a radius of 46. Novoindex (2.8) was used to create a transcriptome index using the combined splice junction and hg19 chromosome sequences.

Reads were aligned to the transcriptome index described above using Novoalign (v2.08.01), allowing up to 50 alignments for each read. USeq (v8.8.2) SamTranscriptomeParser was used to convert the coordinates of reads aligning to splice junctions to genomic space. Reads aligning to multiple locations in the genome with equal confidence were discarded.

Ensembl Build 74 transcript annotations were collapsed into gene annotations using USeq (v8.8.2) MergeUCSCGeneTable. USeq (v8.8.8) DefinedRegionDifferentialSeq was used to generate read counts for each gene. DESeq2 was used to test for differential expression in any of the genotypes using the likelihood ratio test (LRT) option [40]. Genes with significant adjusted p-values were used in Ingenuity Pathway Analysis (IPA^®^, Ingenuity Systems, QIAGEN) and for eQTL expression analysis. For eQTL expression analysis, all genes within an LD block defined by an r^2^ = 0.1 were considered. In addition, expression in fat and skin for all genes within 1 MB of the variant of interest were examined, which encompassed the LD block defined above. Pathway analysis was performed using IPA^®^, which creates algorithmically generated pathways from expression data and compares the ratio of overlap to known pathways. The upstream regulator data is generated by determining differentially expressed genes between genotypes and comparing them to genes regulated by the upstream molecules to determine the significance of enrichment of the gene expression data for the genes downstream of an upstream regulator. For both pathway and upstream regulator analysis, significance is calculated using the Fisher exact test.

## Supporting Information

S1 Table Upstream regulators identified in adipose and skin at gene loci associated with risk for polycystic ovary syndrome. (XLSX)

S2 Table Top five canonical pathways identified by gene expression patterns at each polycystic ovary syndrome genetic risk locus. (XLSX)

S3 Table Gene variants associated with polycystic ovary syndrome risk. (DOCX)

## References

[1] Revised 2003 consensus on diagnostic criteria and long-term health risks related to polycystic ovary syndrome (PCOS). Hum Reprod 2004; 19:41–47 14688154 10.1093/humrep/deh098

[2] Ehrmann DA, Barnes RB, Rosenfield RL. Polycystic ovary syndrome as a form of functional ovarian hyperandrogenism due to dysregulation of androgen secretion. Endo Rev 1995; 16:322–353 10.1210/edrv-16-3-322 7671850

[3] Ehrmann DA, Barnes RB, Rosenfield RL, Cavaghan MK, Imperial J. Prevalence of impaired glucose tolerance and diabetes in women with polycystic ovary syndrome. Diabetes Care 1999; 22:141–146 10333916 10.2337/diacare.22.1.141

[4] Vink JM, Sadrzadeh S, Lambalk CB, Boomsma DI. Heritability of polycystic ovary syndrome in a Dutch twin-family study. J Clin Endocrinol Metab 2006; 91:2100–2104 10.1210/jc.2005-1494 16219714

[5] Shi Y, Zhao H, Shi Y, Cao Y, Yang D, Li Z, Zhang B, Liang X, Li T, Chen J, Shen J, Zhao J, You L, Gao X, Zhu D, Zhao X, Yan Y, Qin Y, Li W, Yan J, Wang Q, Zhao J, Geng L, Ma J, Zhao Y, He G, Zhang A, Zou S, Yang A, Liu J, Li W, Li B, Wan C, Qin Y, Shi J, Yang J, Jiang H, Xu JE, Qi X, Sun Y, Zhang Y, Hao C, Ju X, Zhao D, Ren CE, Li X, Zhang W, Zhang Y, Zhang J, Wu D, Zhang C, He L, Chen ZJ. Genome-wide association study identifies eight new risk loci for polycystic ovary syndrome. Nat Genet 2012; 44:1020–1025 10.1038/ng.2384 22885925

[6] Chen ZJ, Zhao H, He L, Shi Y, Qin Y, Shi Y, Li Z, You L, Zhao J, Liu J, Liang X, Zhao X, Zhao J, Sun Y, Zhang B, Jiang H, Zhao D, Bian Y, Gao X, Geng L, Li Y, Zhu D, Sun X, Xu JE, Hao C, Ren CE, Zhang Y, Chen S, Zhang W, Yang A, Yan J, Li Y, Ma J, Zhao Y. Genome-wide association study identifies susceptibility loci for polycystic ovary syndrome on chromosome 2p16.3, 2p21 and 9q33.3. Nat Genet 2011; 43:55–59 10.1038/ng.732 21151128

[7] Day FR, Hinds DA, Tung JY, Stolk L, Styrkarsdottir U, Saxena R, Bjonnes A, Broer L, Dunger DB, Halldorsson BV, Lawlor DA, Laval G, Mathieson I, McCardle WL, Louwers Y, Meun C, Ring S, Scott RA, Sulem P, Uitterlinden AG, Wareham NJ, Thorsteinsdottir U, Welt C, Stefansson K, Laven JS, Ong KK, Perry JR. Causal mechanisms and balancing selection inferred from genetic associations with polycystic ovary syndrome. Nat Commun 2015; 6:8464 10.1038/ncomms9464 26416764 PMC4598835

[8] Meun CK, T.;Magi,R.;Drong,A.W.;Armstrong,L.;Broer,L.;Jones,M.R.;Day,F.R.;PCOS Genetics Consortium. Program #933: Genome-wide meta-analysis of polycystic ovary syndrome in women of European ancestry identifies new loci in hormone pathways. Presented at the 65th Annual meeting of the American Society of Human Genetics October 7, 2015; Baltimore, MD

[9] Hayes MG, Urbanek M, Ehrmann DA, Armstrong LL, Lee JY, Sisk R, Karaderi T, Barber TM, McCarthy MI, Franks S, Lindgren CM, Welt CK, Diamanti-Kandarakis E, Panidis D, Goodarzi MO, Azziz R, Zhang Y, James RG, Olivier M, Kissebah AH, Reproductive Medicine N, Stener-Victorin E, Legro RS, Dunaif A. Genome-wide association of polycystic ovary syndrome implicates alterations in gonadotropin secretion in European ancestry populations. Nat Commun 2015; 6:7502 10.1038/ncomms8502 26284813 PMC4557132

[10] Welt CK, Styrkarsdottir U, Ehrmann DA, Thorleifsson G, Arason G, Gudmundsson JA, Ober C, Rosenfield RL, Saxena R, Thorsteinsdottir U, Crowley WF, Stefansson K. Variants in DENND1A are associated with polycystic ovary syndrome in women of European ancestry. J Clin Endocrinol Metab 2012; 97:E1342–1347 10.1210/jc.2011-3478 22547425 PMC3387396

[11] van de Bunt M, Manning Fox JE, Dai X, Barrett A, Grey C, Li L, Bennett AJ, Johnson PR, Rajotte RV, Gaulton KJ, Dermitzakis ET, MacDonald PE, McCarthy MI, Gloyn AL. Transcript Expression Data from Human Islets Links Regulatory Signals from Genome-Wide Association Studies for Type 2 Diabetes and Glycemic Traits to Their Downstream Effectors. PLoS Genet 2015; 11:e1005694 10.1371/journal.pgen.1005694 26624892 PMC4666611

[12] Grundberg E, Small KS, Hedman AK, Nica AC, Buil A, Keildson S, Bell JT, Yang TP, Meduri E, Barrett A, Nisbett J, Sekowska M, Wilk A, Shin SY, Glass D, Travers M, Min JL, Ring S, Ho K, Thorleifsson G, Kong A, Thorsteindottir U, Ainali C, Dimas AS, Hassanali N, Ingle C, Knowles D, Krestyaninova M, Lowe CE, Di Meglio P, Montgomery SB, Parts L, Potter S, Surdulescu G, Tsaprouni L, Tsoka S, Bataille V, Durbin R, Nestle FO, O'Rahilly S, Soranzo N, Lindgren CM, Zondervan KT, Ahmadi KR, Schadt EE, Stefansson K, Smith GD, McCarthy MI, Deloukas P, Dermitzakis ET, Spector TD, Multiple Tissue Human Expression Resource C. Mapping cis- and trans-regulatory effects across multiple tissues in twins. Nat Genet 2012; 44:1084–1089 10.1038/ng.2394 22941192 PMC3784328

[13] Small KS, Hedman AK, Grundberg E, Nica AC, Thorleifsson G, Kong A, Thorsteindottir U, Shin SY, Richards HB, Consortium G, Investigators M, Consortium D, Soranzo N, Ahmadi KR, Lindgren CM, Stefansson K, Dermitzakis ET, Deloukas P, Spector TD, McCarthy MI, Mu TC. Identification of an imprinted master trans regulator at the KLF14 locus related to multiple metabolic phenotypes. Nat Genet 2011; 43:561–564 10.1038/ng.833 21572415 PMC3192952

[14] Fairfax BP, Humburg P, Makino S, Naranbhai V, Wong D, Lau E, Jostins L, Plant K, Andrews R, McGee C, Knight JC. Innate immune activity conditions the effect of regulatory variants upon monocyte gene expression. Science 2014; 343:1246949 10.1126/science.1246949 24604202 PMC4064786

[15] Saxena R, Bjonnes AC, Georgopoulos NA, Koika V, Panidis D, Welt CK. Gene variants associated with age at menopause are also associated with polycystic ovary syndrome, gonadotrophins and ovarian volume. Hum Reprod 2015; 30:1697–1703 10.1093/humrep/dev110 25994816 PMC4472323

[16] Saxena R, Bjonnes AC, Georgopoulos NA, Koika V, Panidis D, Welt CK. Gene variants associated with age at menopause are also associated with polycystic ovary syndrome, gonadotrophins and ovarian volume. Human reproduction. 2015;30(7):1697–703. 10.1093/humrep/dev110 25994816 PMC4472323

[17] Ruth KS, Campbell PJ, Chew S, Lim EM, Hadlow N, Stuckey BG, Brown SJ, Feenstra B, Joseph J, Surdulescu GL, Zheng HF, Richards JB, Murray A, Spector TD, Wilson SG, Perry JR. Genome-wide association study with 1000 genomes imputation identifies signals for nine sex hormone-related phenotypes. Eur J Hum Genet 2015; 10.1038/ejhg.2015.102 PMC4564946 26014426

[18] Abel MH, Widen A, Wang X, Huhtaniemi I, Pakarinen P, Kumar TR, Christian HC. Pituitary Gonadotrophic Hormone Synthesis, Secretion, Subunit Gene Expression and Cell Structure in Normal and Follicle-Stimulating Hormone beta Knockout, Follicle-Stimulating Hormone Receptor Knockout, Luteinising Hormone Receptor Knockout, Hypogonadal and Ovariectomised Female Mice. J Neuroendocrinol 2014; 26:785–795 10.1111/jne.12178 25039914 PMC5604239

[19] Fortin J, Boehm U, Deng CX, Treier M, Bernard DJ. Follicle-stimulating hormone synthesis and fertility depend on SMAD4 and FOXL2. FASEB J 2014; 28:3396–3410 10.1096/fj.14-249532 24739304 PMC4101660

[20] Luftner D, Jung A, Schmid P, Geppert R, Kienle E, Wernecke KD, Possinger K, Takeda Adjuvant Breast Cancer Study with Leuprorelin Study G. Upregulation of HER-2/neu by ovarian ablation: results of a randomized trial comparing leuprorelin to CMF as adjuvant therapy in node-positive breast cancer patients. Breast Cancer Res Treat 2003; 80:245–255 14503797 10.1023/a:1024911625339

[21] Goodarzi MO, Jones MR, Li X, Chua AK, Garcia OA, Chen YD, Krauss RM, Rotter JI, Ankener W, Legro RS, Azziz R, Strauss JF 3rd, Dunaif A, Urbanek M. Replication of association of DENND1A and THADA variants with polycystic ovary syndrome in European cohorts. J Med Genet 2012; 49:90–95 10.1136/jmedgenet-2011-100427 22180642 PMC3536488

[22] Pau CT, Keefe C, Duran J, Welt CK. Metformin improves glucose effectiveness, not insulin sensitivity: predicting treatment response in women with polycystic ovary syndrome in an open-label, interventional study. J Clin Endocrinol Metab 2014; 99:1870–1878 10.1210/jc.2013-4021 24606093 PMC4010712

[23] Simonis-Bik AM, Nijpels G, van Haeften TW, Houwing-Duistermaat JJ, Boomsma DI, Reiling E, van Hove EC, Diamant M, Kramer MH, Heine RJ, Maassen JA, Slagboom PE, Willemsen G, Dekker JM, Eekhoff EM, de Geus EJ, t Hart LM. Gene variants in the novel type 2 diabetes loci CDC123/CAMK1D, THADA, ADAMTS9, BCL11A, and MTNR1B affect different aspects of pancreatic beta-cell function. Diabetes 2010; 59:293–301 10.2337/db09-1048 19833888 PMC2797936

[24] Borowiec M, Liew CW, Thompson R, Boonyasrisawat W, Hu J, Mlynarski WM, El Khattabi I, Kim SH, Marselli L, Rich SS, Krolewski AS, Bonner-Weir S, Sharma A, Sale M, Mychaleckyj JC, Kulkarni RN, Doria A. Mutations at the BLK locus linked to maturity onset diabetes of the young and beta-cell dysfunction. PNAS 2009; 106:14460–14465 10.1073/pnas.0906474106 19667185 PMC2732833

[25] Chakraborty A, Wakamiya M, Venkova-Canova T, Pandita RK, Aguilera-Aguirre L, Sarker AH, Singh DK, Hosoki K, Wood TG, Sharma G, Cardenas V, Sarkar PS, Sur S, Pandita TK, Boldogh I, Hazra TK. Neil2-null Mice Accumulate Oxidized DNA Bases in the Transcriptionally Active Sequences of the Genome and Are Susceptible to Innate Inflammation. J Biol Chem 2015; 290:24636–24648 10.1074/jbc.M115.658146 26245904 PMC4598976

[26] Toyoshima Y, Karas M, Yakar S, Dupont J, Lee H, LeRoith D. TDAG51 mediates the effects of insulin-like growth factor I (IGF-I) on cell survival. J Biol Chem 2004; 279:25898–25904 10.1074/jbc.M400661200 15037619

[27] Poretsky L, Cataldo NA, Rosenwaks Z, Giudice LC. The insulin-related ovarian regulatory system in health and disease. Endo Rev 1999; 20:535–582 10.1210/edrv.20.4.0374 10453357

[28] Gibbs GM, Lo JC, Nixon B, Jamsai D, O'Connor AE, Rijal S, Sanchez-Partida LG, Hearn MT, Bianco DM, O'Bryan MK. Glioma pathogenesis-related 1-like 1 is testis enriched, dynamically modified, and redistributed during male germ cell maturation and has a potential role in sperm-oocyte binding. Endocrinology 2010; 151:2331–2342 10.1210/en.2009-1255 20219979

[29] Thackray VG, Mellon PL, Coss D. Hormones in synergy: regulation of the pituitary gonadotropin genes. Mol Cell Endocrinol 2010; 314:192–203 10.1016/j.mce.2009.09.003 19747958 PMC2815122

[30] Raja-Khan N, Urbanek M, Rodgers RJ, Legro RS. The role of TGF-beta in polycystic ovary syndrome. Reprod Sciences 2014; 21:20–31 10.1177/1933719113485294 PMC5933191 23585338

[31] Jones MR, Brower MA, Xu N, Cui J, Mengesha E, Chen YD, Taylor KD, Azziz R, Goodarzi MO. Systems Genetics Reveals the Functional Context of PCOS Loci and Identifies Genetic and Molecular Mechanisms of Disease Heterogeneity. PLoS Genet 2015; 11:e1005455 10.1371/journal.pgen.1005455 26305227 PMC4549292

[32] Bhatt S, Mutharasan P, Garcia OA, Jafari N, Legro RS, Dunaif A, Urbanek M. The inflammatory gene pathway is not a major contributor to polycystic ovary snydrome. J Clin Endocrinol Metab 2014; 99:E567–571 10.1210/jc.2013-2342 24423322 PMC3942235

[33] Carmina E, Chu MC, Moran C, Tortoriello D, Vardhana P, Tena G, Preciado R, Lobo R. Subcutaneous and omental fat expression of adiponectin and leptin in women with polycystic ovary syndrome. Fertil Steril 2008; 89:642–648 10.1016/j.fertnstert.2007.03.085 17562334

[34] Martinez-Garcia MA, Montes-Nieto R, Fernandez-Duran E, Insenser M, Luque-Ramirez M, Escobar-Morreale HF. Evidence for masculinization of adipokine gene expression in visceral and subcutaneous adipose tissue of obese women with polycystic ovary syndrome (PCOS). J Clin Endocrinol Metab 2013; 98:E388–396 10.1210/jc.2012-3414 23337724

[35] Manneras-Holm L, Leonhardt H, Kullberg J, Jennische E, Oden A, Holm G, et al Adipose tissue has aberrant morphology and function in PCOS: enlarged adipocytes and low serum adiponectin, but not circulating sex steroids, are strongly associated with insulin resistance. The Journal of clinical endocrinology and metabolism. 2011;96:E304–11 10.1210/jc.2010-1290 21084397

[36] Welt CK, Gudmundsson JA, Arason G, Adams J, Palsdottir H, Gudlaugsdottir G, et al Characterizing discrete subsets of polycystic ovary syndrome as defined by the Rotterdam criteria: the impact of weight on phenotype and metabolic features. The Journal of clinical endocrinology and metabolism. 2006;91:4842–8 10.1210/jc.2006-1327 17003085

[37] Welt CK, Arason G, Gudmundsson JA, Adams J, Palsdottir H, Gudlaugsdottir G, et al Defining constant versus variable phenotypic features of women with polycystic ovary syndrome using different ethnic groups and populations. The Journal of clinical endocrinology and metabolism. 2006;91:4361–8 10.1210/jc.2006-1191 16940441

[38] Taylor AE, McCourt B, Martin KA, Anderson EJ, Adams JM, Schoenfeld D, et al Determinants of abnormal gonadotropin secretion in clinically defined women with polycystic ovary syndrome. The Journal of clinical endocrinology and metabolism. 1997;82:2248–56 10.1210/jcem.82.7.4105 9215302

[39] Barrett JC, Fry B, Maller J, Daly MJ. Haploview: analysis and visualization of LD and haplotype maps. Bioinformatics. 2005;21:263–5 10.1093/bioinformatics/bth457 15297300

[40] Love MI, Huber W, Anders S. Moderated estimation of fold change and dispersion for RNA-seq data with DESeq2. Genome Biol. 2014;15:550 10.1186/s13059-014-0550-8 25516281 PMC4302049

