# Supplementary material for: Phenotype and Tissue Expression as a Function of Genetic Risk in Polycystic Ovary Syndrome
Source: PLoS One. 2017 Jan 9;12(1):e0168870. doi: 10.1371/journal.pone.0168870 (PMC5221814; doi:10.1371/journal.pone.0168870)
Supplement: S3 Table — (DOCX) [file pone.0168870.s003.docx]

Supplementary Table 3. Gene variants associated with polycystic ovary syndrome risk. Fifteen have been identified or replicated in studies of women of European ethnicity with nominal significance with the exception of rs6022786.

| Variant | Chr | Location | Nearest Gene | Distance* | RSquared | DPrime | Reference |
| --- | --- | --- | --- | --- | --- | --- | --- |
| rs13429458 | 2 | 43638838 | *LHCGR* |  |  |  | [1] |
| rs12478601 | 2 | 43721508 | *THADA* |  |  |  | [1] |
| rs2268361 | 2 | 49201612 | *FSHR* |  |  |  | [2] |
| rs2178575 | 2 | 213391766 | *ERBB4* |  |  |  | [3]# |
| rs13164856 | 5 | 131813204 | *RAD50/IRF1* |  |  |  | [3]# |
| rs804279 | 8 | 11766130 | *GATA4* |  |  |  | [4]# |
| rs4385527 | 9 | 126525212 | *C9orf3* |  |  |  | [2, 4] |
| rs2479106 | 9 | 126525212 | *DENND1A* | 24743 | 0.105 | 1 | [1] |
| rs10986105 | 9 | 126549955 | *DENND1A* | 24743 | 0.105 | 1 | [1] |
| rs11031005 | 11 | 30226356 | *FSHB* | 172 | 1 | 1 | [3, 4] |
| rs11031006 | 11 | 30226528 | *FSHB* | 172 | 1 | 1 | [3, 4] |
| rs1894116 | 11 | 102070639 | *YAP1* |  |  |  | [2] |
| rs705702 | 12 | 56390636 | *RAB5b* | 87058 | 0.466 | 0.805 | [2] |
| rs2271194 | 12 | 56411694 | *ERBB3* | 87058 | 0.466 | 0.805 | [3]# |
| rs2272046 | 12 | 66224461 | *HMG2A* |  |  |  | [2] |
| rs1795379 | 12 | 75941042 | *KRR1* |  |  |  | [3]# |
| rs4784165 | 16 | 52347819 | *TOX3* |  |  |  | [2] |
| rs2059807 | 19 | 7166109 | *INSR* |  |  |  | [2] |
| rs6022786 | 20 | 52447303 | *SUMO1P* |  |  |  | [2] |

*For variants at the same gene locus, the distance between the variants, the r^2^ and D prime are presented to demonstrate the degree of linkage disequilibrium between the two variants.

#Phenotype not examined previously in the current cohort.

1. Chen ZJ, Zhao H, He L, Shi Y, Qin Y, Shi Y, et al. Genome-wide association study identifies susceptibility loci for polycystic ovary syndrome on chromosome 2p16.3, 2p21 and 9q33.3. Nature genetics. 2011;43(1):55-9. doi: 10.1038/ng.732. PubMed PMID: 21151128.

2. Shi Y, Zhao H, Shi Y, Cao Y, Yang D, Li Z, et al. Genome-wide association study identifies eight new risk loci for polycystic ovary syndrome. Nature genetics. 2012;44(9):1020-5. doi: 10.1038/ng.2384. PubMed PMID: 22885925.

3. Day FR, Hinds DA, Tung JY, Stolk L, Styrkarsdottir U, Saxena R, et al. Causal mechanisms and balancing selection inferred from genetic associations with polycystic ovary syndrome. Nat Commun. 2015;6:8464. doi: 10.1038/ncomms9464. PubMed PMID: 26416764; PubMed Central PMCID: PMCPMC4598835.

4. Hayes MG, Urbanek M, Ehrmann DA, Armstrong LL, Lee JY, Sisk R, et al. Genome-wide association of polycystic ovary syndrome implicates alterations in gonadotropin secretion in European ancestry populations. Nat Commun. 2015;6:7502. doi: 10.1038/ncomms8502. PubMed PMID: 26284813; PubMed Central PMCID: PMCPMC4557132.
